# Fatal Retroperitoneal Bleeding Caused by Neurofibromatosis: A Case Report and Review of the Literature

**DOI:** 10.1155/2015/965704

**Published:** 2015-01-26

**Authors:** Patrick R. Moerbeek, Jesse M. van Buijtenen, Baukje van den Heuvel, Arjan W. J. Hoksbergen

**Affiliations:** Department of Surgery, VU University Medical Center, P.O. Box 7057, 1007 MB Amsterdam, Netherlands

## Abstract

A young female was brought into the emergency department with pulseless electrical activity (PEA) after local resection of neurofibromateous lesions. Chest ultrasonography was normal. Abdominal ultrasonography was not performed. After successful resuscitation a total body CT-scan was performed to rule out potential bleeding sources. However, haemodynamic instability reoccurred and the scan had to be aborted at the thoracoabdominal level. No thoracic abnormalities were found. Resuscitation was reinitiated and abdominal ultrasonography was performed, showing a large amount of abdominal fluid. A progressive fall in haemoglobin was noted. Emergency laparotomy was performed, revealing a large retroperitoneal haematoma. Despite ligation and packing, bleeding continued. Postoperative angiography showed active bleeding from a branch of the left internal iliac artery, which could be successfully coiled. Unfortunately, the patient died five days later due to irreversible brain damage. Revision of an MRI scan made one year earlier showed a 10 cm large retroperitoneal neurofibromatous lesion exactly at the location of the current bleeding. This case shows that patients with neurofibromatosis might develop spontaneous life-threatening bleeding from retroperitoneal located lesions. Furthermore, it points out the necessity of focused assessment with ultrasonography of the abdomen in all patients with PEA of unknown origin.

## 1. Case Report

A 33-year-old female with a medical history of neurofibromatosis type 1 and scoliosis was presented to our emergency department with PEA. She collapsed at home after complaints of “heavy arms” and nausea some hours after local excision of numerous cutaneous neurofibromatous lesions in another hospital. Surgery had been performed under general anaesthesia and local application of ropivacaine (a local anaesthetic of the amide type). At arrival of the paramedics asystole was found and immediate resuscitation was started, regaining cardiac output and blood pressure. During transport cardiac arrest reoccurred, forcing the ambulance staff to divert her to the nearest hospital.

After regaining output, sinus rhythm of 70 beats/min with blood pressure of 80/60 mmHg was established. The initial lactate was 21 mmol/L, glucose 31 mmol/L, and haemoglobin 6.0 mmol/L. Arterial blood gasses showed a pH 6.5 mmol/L, potassium 5.5 mmol/L,* p*O_2_ 287 mmHg, and* s*O_2_ of 98%.

The initial consideration was anaphylactic shock caused by the local anaesthetics, diabetic ketoacidosis de novo, or pulmonary embolism. Immediate chest ultrasonography showed no signs of pulmonary embolism or cardiac tamponade. Since the surgery earlier that day involved only cutaneous resections and there had been no abdominal complaints, an abdominal cause was not immediately suspected.

After stabilizing the patient a total-body CT-scan was made to rule out potential bleeding sources. However, during this process haemodynamic instability reoccurred requiring instant resuscitation and transport to the intensive care unit (ICU). The CT-scan had to be aborted at the diaphragm level and showed no intracerebral or thoracic abnormalities.

During the second resuscitation a significant fall in haemoglobin was noted. Abdominal ultrasonography now showed a large amount of abdominal fluid and clots. Laceration of the liver or spleen due to the chest compressions during resuscitation was suspected and because of ongoing hemodynamic instability immediate laparotomy was performed. A large expanding retroperitoneal haematoma in the left lower quadrant of the abdomen was identified and explored showing a bleeding at the level of the lumbar vertebrae. Attempts were made to control the haemorrhage by suturing, application of local haemostatics, and gauze packing.

**Figure 1:**
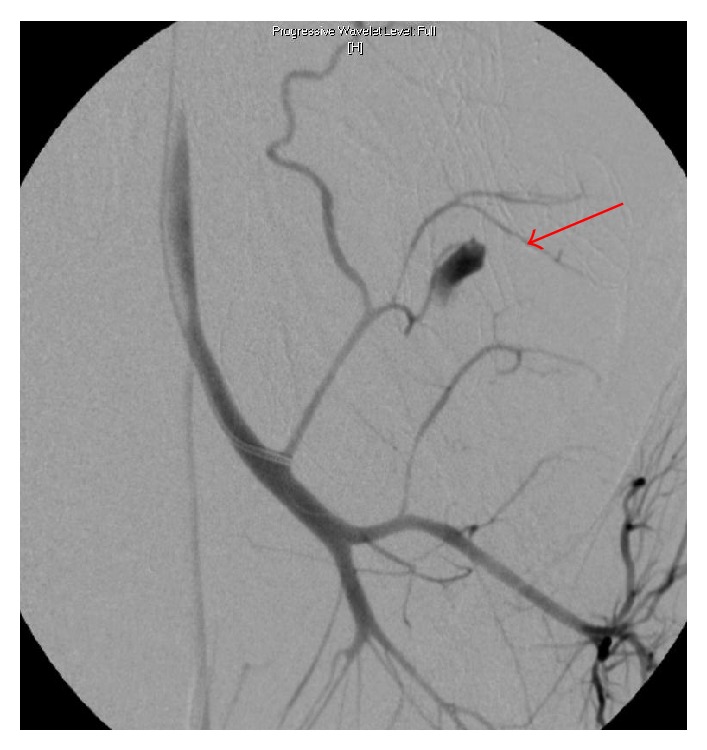
Angiography showing a blush from a dorsal branch from the left internal iliac artery (arrow).

**Figure 2:**
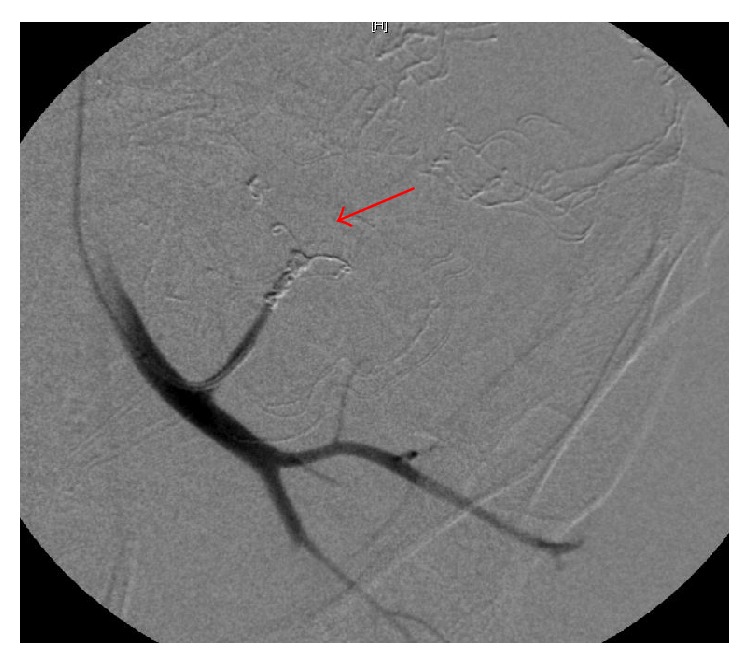
Angiography showing successful coiling of the bleeding artery.

Despite these efforts, the bleeding persisted postoperatively and angiography was performed. Contrast extravasation from a dorsal branch of the left internal iliac artery was identified and was successfully coiled (Figures 1 and 2). After this procedure the patient stabilized and was transferred to the ICU.

In total 31 units of packed cells, 22 units of fresh frozen plasma cells, and 7 units of thrombocytes were infused. Unfortunately 5 days later the patient died because of severe irreversible ischemic brain damage. Autopsy was declined by the family.

**Figure 3:**
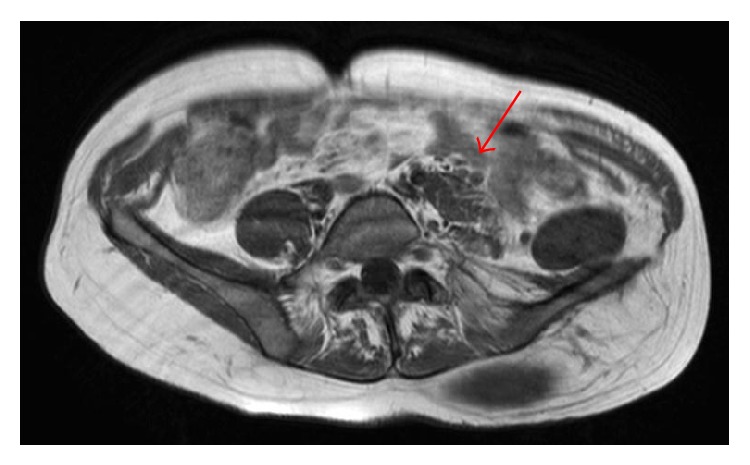
MRI scan showing the retroperitoneal NF1 lesion (arrow) at the bleeding site. Lateral displacement of left psoas muscle due to tumour growth.

After her death an MRI scan made one year earlier in the hospital where she was known was revised and revealed a 10 cm large neurofibromatous lesion exactly at the location of the present haemorrhage (Figure 3). This knowledge was unfortunately not available at the time of presentation.

## 2. Discussion

Neurofibromatosis (NF) is a genetically autosomal dominant inherited condition in which tumours grow from nerve tissue [1]. There are two types of NF. NF type I, also known as Von Recklinghausen's disease, causes mostly neurocutaneous lesions. It is also the most common type, accounting for 90% of all neurofibromatosis cases, with an incidence of 1 in 3000. NF type II is seen in the central nerve system and is less common with an incidence of 1 in 45000. The clinical manifestations of NF1 are extremely variable. The hallmarks of NF1 are multiple “café-au-lait” spots and associated cutaneous neurofibromas. Four types of neurofibromas can be distinguished mainly based on their origin: cutaneous, subcutaneous, nodular, and diffuse plexiform types.

The diffuse plexiform type of neurofibromatosis is the most vascularized type. In 5–15% the benign neurofibromas may convert to malignant tumours [2]. Up to 25% of NF1 patients develop gastrointestinal stromal tumours (GISTs) with nonspecific presentations [3]. There is no specific treatment for NF1; however monitoring is advised. Lesions may be resected if desirable [4].

Vascular manifestations of NF1, such as occlusion, stenosis, aneurysm, and ectasia, were first described in 1945 [5]. The incidence of vascular involvement of NF1, mainly stenosis and aneurysms, is approximately 3.6% [6]. Arterial rupture, fistulas, and occlusion are rare and are presumably caused by arterial fragility secondary to arterial dysplasia or vascular invasion by the neurofibroma [5, 7]. Vasculopathy can range from large vessels in visceral organs to small vessels in soft tissue [8].

A review of the English literature from 1957 to 2007 by Oderich et al. showed 237 patients with NF1 with 320 vascular abnormalities [9]. This number, however, is probably underestimated, as most of the lesions are clinically silent. Arterial thickening, stenosis, and aneurysms are frequently seen in postmortal examinations [10]. Haemorrhage caused by neurofibromatosis can be severe. Several cases have been described. The sites of bleeding most frequently seen are intrathoracic [1], intraluminal (GISTs) [3], and subcutaneous [11]. Cerebral, organic, and retroperitoneal haemorrhages are less commonly described.

Vascular involvement is considered to be one of the most important causes of early death in patients with NF1 [7, 12–14]. Most vascular lesions are diagnosed after complaints of pain or haemorrhage and are found at a mean age of the patients of 38 (±16 years).

Diagnosis of spontaneous abdominal or retroperitoneal haemorrhage is often delayed or missed. When such a bleeding occurs from NF1, patients may rapidly develop hypovolemic shock. Life expectancy in NF1 patients is 15 years shorter with a mean age of 59 years. After malignancy, bleeding is the second cause of early death especially in patients under the age of 40 [9].

Unfamiliarity with the pathophysiology of NF1 may lead to delayed diagnosis and treatment in patients presenting with hypovolemic shock. Focused assessment with sonography of the abdomen, which can easily be performed in the emergency room during initial workup, is a fast and helpful tool to identify abdominal haemorrhage and facilitate decision-making [15].

Since haemorrhage is typically seen in NF1 patients in their 3rd and 4th decades, screening for neurofibromas with possible vascular involvement in this group seems opportune. Also the potential malignant conversion may warrant screening and frequent follow-up in this particular group. However, Wolkenstein et al. [16] reported minimal benefits of screening investigations solely based on chest X-rays and abdominal ultrasonography. Nowadays low-dose CT-scanning and MRI are widely available and sensitivity and specificity are superior to ultrasound. In this way lesions with vascular involvement might be identified in an earlier stage and sudden, potentially lethal, haemorrhage might be prevented.

Several cases describe successful endovascular treatment of acute haemorrhage in NF1, even from multiple bleeding sites [11, 13, 17–19]. Failure after coiling, however, has been described [20].

Treatment of retroperitoneal bleeding in hemodynamically unstable patients remains challenging. Whether patients are treated best with angiography and coiling and/or surgical packing depends on several factors including haemodynamic instability, diagnosis, bleeding site, and local expertise and experience.

## 3. Conclusion

Neurofibromatous lesions can be located intra-abdominally or retroperitoneally and may cause serious bleeding due to vascular involvement, especially in relatively young patients. In patients with a history of neurofibromatosis who present with sudden collapse and pulseless electrical activity, urgent identification of possible abdominal haemorrhage is essential. Focused assessment with sonography during initial workup in such patients is a fast and helpful tool to identify an abdominal source of bleeding.

## References

[1] Hongsakul K., Rookkapan S., Tanutit P., Pakdeejit S., Songjamrat A., Sungsiri J. (2013). Spontaneous massive hemothorax in a patient with neurofibromatosis type 1 with successful transarterial embolization. *Korean Journal of Radiology*.

[2] Bakker J. R., Haber M. M., Garcia F. U. (2005). Gastrointestinal neurofibromatosis: an unusual cause of gastric outlet obstruction. *The American Surgeon*.

[3] Kupec J. T., Williams H. J., Roorda A. K., Goebel S. U. (2011). Recurrent hematochezia secondary to gastrointestinal stromal tumors (GISTs) in neurofibromatosis type one. *The West Virginia Medical Journal*.

[4] http://www.uptodate.com.

[5] Reubi F. (1945). Neurofibromatose et lesions vasculaires. *Schweizerische Medizinische Wochenschrift*.

[6] Brasfield R. D., Das Gupta T. K. (1972). Von Recklinghausen's disease: a clinicopathological study. *Annals of Surgery*.

[7] Friedman J. M., Arbiter J., Epstein J. A. (2002). Cardiovascular disease in neurofibromatosis 1: report of the NF1 cardiovascular task force. *Genetics in Medicine*.

[8] Jett K., Friedman J. M. (2010). Clinical and genetic aspects of neurofibromatosis 1. *Genetics in Medicine*.

[9] Oderich G. S., Sullivan T. M., Bower T. C. (2007). Vascular abnormalities in patients with neurofibromatosis syndrome type I: clinical spectrum, management, and results. *Journal of Vascular Surgery*.

[10] Racacardi V. M. (1992). *Neurofibromatosis: Phenotype, Natural History and Pathogenesis*.

[11] Zhang K., Song J., Xiong W. (2012). Massive spontaneous hemorrhage in giant type 1 neurofibromatosis in soft tissue of chest wall. *The Journal of Thoracic and Cardiovascular Surgery*.

[12] Conlon N. P., Redmond K. C., Celi L. A. (2007). Spontaneous hemothorax in a patient with neurofibromatosis type I and undiagnosed pheochromocytoma. *Annals of Thoracic Surgery*.

[13] Kim H. J., Seon H. J., Choi S., Jang N. K. (2011). Ruptured aneurysm of intercostal arteriovenous malformation associated with neurofibromatosis type 1: a case report. *CardioVascular and Interventional Radiology*.

[14] Pomara C., Bello S., D'Errico S., Greco M., Fineschi V. (2013). Sudden death due to a dissecting intramural hematoma of the esophagus (DIHE) in a woman with severe neurofibromatosis-related scoliosis. *Forensic Science International*.

[15] Fleming S., Bird R., Ratnasingham K., Sarker S.-J., Walsh M., Patel B. (2012). Accuracy of FAST scan in blunt abdominal trauma in a major London trauma centre. *International Journal of Surgery*.

[16] Wolkenstein P., Frèche B., Zeller J., Revuz J. (1996). Usefulness of screening investigations in neurofibromatosis type 1: a study of 152 patients. *Archives of Dermatology*.

[17] Misao T., Yoshikawa T., Aoe M., Ueda Y., Yodoya M., Sakurai J. (2012). Recurrent rupture of intercostal artery aneurysms in neurofibromatosis type 1. *General Thoracic and Cardiovascular Surgery*.

[18] Choong A., Alagaratnam S., Suliman S. (2012). The endovascular management of a neurofibromatosis vasculopathy: a case report. *Vascular and Endovascular Surgery*.

[19] Hung M. C., Yang E., Huang Y. C., Chang R.-S. (2012). Spontaneous hemorrhage within the neck of a neurofibromatosis type 1 patient. *Journal of Emergency Medicine*.

[20] Vos C. G., Hoksbergen A. W. J. (2011). Fatal retroperitoneal bleeding caused by metastasis of a sigmoid carcinoma. *Case Reports in Medicine*.

